# Assessing the Status of Environmental Education in Illinois Elementary Schools

**DOI:** 10.4137/EHI.S3502

**Published:** 2009-11-09

**Authors:** Rebecca M. Young, Sharron LaFollette

**Affiliations:** Department of Public Health, University of Illinois Springfield, Springfield, IL 62703, USA. Email: slafo1@uis.edu

**Keywords:** environmental education, elementary school, children, survey

## Abstract

One-thousand Illinois elementary teachers received a survey intended to assess the amount and manner in which they included environmental education in the classroom during the 2005 academic year. Over 91% of respondents (n = 234) said that they taught about the environment at least once during the school year, yet most students were only exposed to 22 to 100 minutes during that year. Of the teachers that included environmental education, 49% said they did so because of personal interest in the environment; 47% of the teachers that excluded it said the reason was because of a lack of class time.

Environmental education (EE) is an important component of a child’s education to help him or her develop adequate environmental knowledge and adopt positive attitudes and behaviors^1^,^2^ in order to become an environmentally literate individual.^3^ Environmental education enables children to become socially responsible individuals and make conscientious decisions about the future of the environment.^4^ Teaching children to respect and understand the environment and its associated problems not only contributes to creating socially responsible individuals, but can also help them in their overall educational experience. Students participating in environmental education programs showed improved reading, writing, and oral communication skills.^5^

Although the documented benefits of environmental education are numerous, the extent and nature of environmental education in classrooms throughout the USA are largely unknown. Only one state-wide research study (Wisconsin) on the amount of environmental education existed prior to this study. Lane, Wilke, Champeau, and Sivek^6^ found that 30% of elementary and secondary educators did not teach about the environment, even though Wisconsin requires environmental education at all grade levels.

The state of Illinois recognizes the importance of EE and requires public schools to include instruction, study, and discussion of environmental problems (105 ILCS 5/27-13.1). The legal requirement is loosely reflected in the Illinois Learning Standards issued by the Illinois State Board of Education (ISBE). Recently, the ISBE commissioned the Illinois Environmental Education Advancement Consortium (IEEAC)^7^ to assess the level of EE addressed in the goals of each subject area (language arts, mathematics, science, and social studies). IEEAC determined which goals, when used, could incorporate EE. The science goals appeared to address EE most often. This paper contributes to an improved understanding of environmental education in the State of Illinois by providing empirical data on the time allocated to this subject in elementary schools.

## Method

The purpose of this research was to assess the amount and manner of EE implemented in Illinois and to determine reasons for inclusion and omission of EE. A survey adapted from Lane et al^6^ was distributed in December 2006 to 200 Illinois public elementary schools using proportionate stratified (based on the Illinois Association of Regional Superintendents of Schools’(IARSS))^8^ geographical delineation of the state^a^) random sampling. The authors use this geographical delineation as the state of Illinois greatly varies: while the northeast section of the state is largely urban and suburban (this includes Chicago and surrounding suburbs), the rest of the state is mostly rural. The authors believe that conceptions about the environment may vary between farming communities and non-farming communities.

For the sake of consistency, schools that included only grade levels kindergarten through fifth and that were not magnet or charter schools were eligible for selection.

Because teaching assignments for each school were not easily accessible, surveys were sent to school principals, who were asked in a cover letter to distribute the surveys to teachers at their schools. Each principal was asked to give one survey each to a first, second, third, fourth, and fifth grade teacher. Kindergarten teachers were omitted because their classes sometimes meet for half days allowing for a standardized collection of time spent teaching EE. In addition to the survey, the teachers were given a letter explaining the research and a postage-paid self-addressed return envelope. Teachers were instructed to complete the survey only if they taught at their current grade level during the 2005 to 2006 school year and to supply responses based only on that school year.

While using similar topics used in the Lane et al^6^ survey (incorporation of EE, amount of class time, and teaching methodologies), the present survey did not use a Likert-type instrument employed by Lane et al. Instead, the survey consisted of twelve multiple-choice questions. To assess the level of EE, the survey asked whether or not the teacher included any environmental topics in the classroom, the amount of time teachers spent on those topics, and the manner in which they taught about those topics. Additionally, teachers were asked to indicate their source of environmental information. Teachers were asked why they chose to include or omit environmental information in the classroom and what would encourage them to incorporate more EE. The results were analyzed for differences between grades and geographical regions using the Kruskal-Wallis one-way analysis of variance statistical test.

## Results and Discussion^b^

Of the 1,000 surveys distributed, 256 (25.6%) useable surveys were returned and analyzed (Tables 1 and 2). The majority of respondents (91.4%) indicated that they taught about the environment; the remaining respondents (8.6%) indicated that they did not (Table 3). Although most of the teachers taught about the environment, most of those respondents indicated that they taught it only between two to five times a year (Table 4) for 11 to 20 minutes per lesson (Table 5). Based on those responses, Illinois elementary students may have only been exposed to between 22 to 100 minutes during the 2005 to 2006 school year. With the documented importance of EE and the corresponding educational and social benefits, 22 to 100 minutes may be too little classroom time committed to EE. However, Illinois teachers must follow the curriculum as dictated by the Illinois Learning Standards and their respective districts, which may or may not emphasize EE.

Differences existed in the amount of environmental education presented to students by grade. Fourth grade teachers taught about the environment more frequently than first grade teachers (Table 6), but no differences were present between the other grades or geographically (Table 7). Additionally, environmental lessons taught by fifth grade teachers were longer than those in first or second grades (Table 6), with no differences between the other grades or geographical regions (Table 7). In Illinois, it seems as though the younger children received less EE than the older children. Palmer^9^ called early childhood a “critical time” (p. 388) due to the timing of the younger children formulating thoughts and feelings about the environment. Therefore, Illinois teachers may not be providing opportunities for all students to develop those thoughts and feelings. However, differences in the present study may be due to differences in curricula or Learning Standards for the grades.

### Classroom implementation

An interdisciplinary curricula has been shown to be the most effective method of teaching EE.^4^,^10^,^11^ In Illinois, the majority of the respondents that said they taught about the environment indicated that they most often taught about the environment within science lessons (Table 8). This may also indicate that they were just teaching about the environment and not teaching EE. Science lessons could be a basis for EE but are not the only platform from which to teach it.^3^ Only 18.4% of the Illinois teachers that taught about the environment said that they did so in an interdisciplinary approach. These results imply that Illinois students do not receive the full benefits of an interdisciplinary EE program, and are less likely to develop those critical thinking skills that, according to Paul and Volk,^4^ are important to formulating solutions and making decisions. Also, since only 18.4% of the Illinois teachers included environmental topics into a social studies or social science context, the students were less likely to fully understand the relationship between the environment and social issues that was described by Loughland et al.^11^

A clear majority (88.3%) of Illinois teachers that taught about the environment said that they did so with classroom discussions where the teacher and the students participated (Table 9). According to Basile and White,^10^ classroom discussion is the appropriate teaching method to use in EE programs, instead of relying on instruction or lectures from the teachers.^12^ Very few Illinois teachers selected the other responses about teaching methods such as student or class research projects about environmental problems; going outside on school grounds; field trips to museums, nature centers, or parks; or exploring the students’ environmental values (Table 9). Those teaching methods are all important components of an EE program, especially the firsthand experiences provided by field trips or going outside that help students develop an environmental awareness and concern^1^ and value exploration that is an integral component of EE.^3^

### Reasons for omitting or including EE

Respondents in the present study were asked to indicate their reasons for omitting or including EE. The majority (47.4%) of respondents reporting that they did not teach about the environment selected a lack of class time as the reason (Table 10). In other studies, the most common reasons for omitting EE was that the environment was an unrelated topic^6^ or the teacher lacked the necessary background information to teach about the environment.^4^ In the present study of Illinois teachers, 21.1% of teachers omitting environmental topics said it was because the topics were unrelated; however, only 5.3% said that they did not know enough. As students often are not tested on environmental topics,^12^ teachers may just spend class time on achievement test subjects.

The study showed that the most likely (49.2%) reason that teachers taught about the environment was because of a personal interest in the environment (Table 11). With only 8.6% of teachers indicating that student environmental concern led them to teach about the environment, the need for EE is further highlighted as EE can help students develop concern for the environment that Bogner^1^ discussed. Only three Illinois teachers indicated that they taught about the environment because Illinois law required it. This does not indicate relative teacher knowledge of the law because teachers were only asked to indicate the best reason why they included EE and not every reason.

In the present study, all respondents were asked what would encourage them to include or include more EE. Responses differed greatly based on whether or not the teacher taught about the environment. The most common response among Illinois teachers that included EE was more and easier access to resources (Table 12), while the teachers that omitted EE reported more emphasis from district administrators would encourage them the most (Table 13). However, most of the teachers in the present study reported that a lack of class time limited them from teaching about the environment. It is unlikely that more training or augmented access to resources would solve the problem of limited time. Emphasis from district administrators might overcome that problem, as the emphasis is likely to occur through modified curricula.

## Limitations

### Factors affecting response rate

Even though the response rate for the present study (26.6 percent) appears low when compared with the response rate for the survey (59.2 percent) by Lane et al,^6^ it was much higher than the authors’ expectation of 10 percent as the present survey was performed as part of a graduate research thesis. Several factors may have played a role in the response rate for the present study. Possibly the most important factor is that the surveys were sent in the beginning of December. With the holidays and winter break approaching, teachers may have been too busy to respond. The teachers were told that the survey had to be returned by December 14th (only 10 days after the surveys were mailed). The short time period provided to the teachers may have also affected the response rate. However, the timing and deadline for the survey were necessary to expedite completion of the study. Additionally, principals may not have distributed the surveys to the teachers, so they may not have had the chance to complete the survey.

Mailing a reminder postcard after the original mailing of the surveys might have increased the response rate in the present study. In a review of 183 previously published survey studies, Heberlein and Baumgartner^13^ showed that by increasing the number of contact occurrences when sending self-administered mail surveys, response rates increased (from an average 46.1 percent response rate with one contact to 80.6 percent response rate with three contacts). Yet in the present study, potential survey respondents were contacted only one time. Nonrespondents were not contacted a second time due to financial and time constraints.

The number of nonrespondents in the present study was 73.4 percent. If the would-be responses of the nonrespondents were different from those that provided completed surveys that were analyzed, then a nonresponse bias would be present.^14^ To determine whether differences were present between the nonrespondents and respondents populations, the student demographic characteristics from the 97 schools where at least one teacher returned a survey were compared to the student demographic characteristics of the 102 schools where no teachers returned any surveys. It was found that those populations were statistically similar when compared by Areas (*U* = 4894.5, *n_respondents_* = 97, *n_nonrespondents_* = 102, *p* = 0.875), percentage of low income students (*U* = 4885, *n_respondents_* = 97, *n_nonrespondents_* = 102, *p* = 0.879), and percentage of White students (*U* = 4474, *n_respondents_* = 97, *n_nonrespondents_* = 102, *p* = 0.244). Since the respondent and nonrespondent populations are not significantly different, it is unlikely that a nonresponse bias is present in the present study, but that was not verified, as the nonrespondents were not contacted to complete the survey questionnaire.

### Survey limitations

Teachers were restricted with their responses on the survey, especially for the question regarding the teaching method used. Respondents in the present study were not given the option to select teacher lectures as a possible response for the applicable survey question. Therefore, it is unknown if Illinois teachers included that method of teaching as well. Additionally, the respondents were only allowed to select one response for each question. By restricting the number of selections, the authors do not know whether teachers use multiple teaching methods to incorporate environmental education.

## Conclusions and Recommendations

Even though 91.4% of Illinois teachers responding to the survey said they taught about the environment, the majority of Illinois elementary school students were only exposed to between 22 to 100 minutes of EE during the 2005 to 2006 school year. With the many documented benefits of EE, encompassing educational and environmental sectors, 22 to 100 minutes for an entire school year may not be enough time to help students gain environmental knowledge and foster positive environmental attitudes and behaviors. Additionally, the majority of teachers did not use the recommended interdisciplinary teaching method. The present study only begins to evaluate the quantity and quality of EE in Illinois elementary classrooms. Because the majority of teachers that said that they omitted EE indicated that they did so because of a lack of class time, the Illinois Learning Standards could be modified to clearly incorporate EE into the goals and standards set for Illinois school children. In other words, EE must be paired with and fully integrated into other required subjects. Additionally, teachers must be shown how to successfully accomplish this within their classrooms through preservice courses and in-service training sessions.

## Figures and Tables

**Table 1.** Useable surveys received by grade level taught.

	**1st Grade**	**2nd Grade**	**3rd Grade**	**4th Grade**	**5th Grade**	**Total**
Surveys sent	200	200	200	200	200	1000
Useable surveys received	47	47	52	60	50	256
% of useable surveys received	23.5	23.5	26.0	30.0	25.0	25.6
% of total useable surveys received	18.4	18.4	20.3	23.4	19.5	100

**Table 2.** Useable surveys received by location of school.

	**Area I**	**Area II**	**Area III**	**Area IV**	**Area V**	**Area VI**	**Total**
Surveys sent	685	95	50	95	65	10	1000
Useable surveys received	175	22	9	26	19	5	256
% of useable surveys received	25.5	23.2	18.0	27.4	29.2	50	25.6
% of total useable surveys received	68.4	8.6	3.5	10.2	7.4	2.0	100

**Table 3.** Frequency of inclusion or omission.

**Inclusion?**	**Grade**					**Area**						**Total**	**Total %**
	**1st**	**2nd**	**3rd**	**4th**	**5th**	**I**	**II**	**III**	**IV**	**V**	**VI**		
Yes	45	45	48	54	42	155	20	9	26	19	5	234	91.4
No	2	2	4	6	8	20	2	0	0	0	0	22	8.6
N	47	47	52	60	50	175	22	9	26	19	5	256	100

**Table 4.** Frequency of lessons.

**Frequency**	**Grade**					**Area**						**Total**	**Total %**
	**1st**	**2nd**	**3rd**	**4th**	**5th**	**I**	**II**	**III**	**IV**	**V**	**VI**		
Once/year	7	1	0	2	2	8	0	0	2	2	0	12	5.2
2 to 5 times/year	22	25	26	15	15	71	8	4	15	3	2	103	44.8
6 to 8 times/year	4	9	8	11	8	24	6	1	2	7	0	40	17.4
Once/month	6	3	6	8	5	20	1	2	1	4	0	28	12.2
2 to 3 times/month	4	3	4	6	5	16	2	1	0	1	2	22	9.4
Once/week	1	3	2	6	4	8	2	1	4	0	1	16	7.0
2 to 5 times/week	1	0	2	3	3	5	1	0	2	1	0	9	3.9
>5 times/week	0	0	0	0	0	0	0	0	0	0	0	0	0.0
N	45	44	48	51	42	152	20	9	26	18	5	230	100

**Table 5.** Length of lessons.

**Length**	**Grade**					**Area**						**Total**	**Total %**
	**1st**	**2nd**	**3rd**	**4th**	**5th**	**I**	**II**	**III**	**IV**	**V**	**VI**		
1 to 10 min.	3	1	3	2	2	6	0	1	1	3	0	11	4.7
11 to 20 min.	23	19	14	22	9	52	9	3	13	5	5	87	37.2
21 to 30 min.	12	18	11	13	10	43	5	2	9	5	0	64	27.4
31 to 40 min.	2	5	16	8	9	29	4	1	3	3	0	40	17.1
41 to 50 min.	4	2	2	5	10	17	2	2	0	2	0	23	9.8
51 to 60 min.	1	0	1	3	1	5	0	0	0	1	0	6	2.6
>60 min.	0	0	1	1	1	3	0	0	0	0	0	3	1.3
N	45	45	48	54	42	155	20	9	26	19	5	234	100

**Table 6.** Differences among grade levels.

**Question number**	**H**	***d_f_***	***p*^a^,^b^**
Q.3 Frequency of lessons	14.171	4	0.007*
Q.4 Length of lessons	14.562	4	0.006*
Q.5 Subject	9.486	4	0.050*
Q.6 Teaching method	1.278	4	0.865
Q.7 Global/Local issues	12.177	4	0.016*
Q.8 Where teacher obtained information	0.299	4	0.990
Q.9 Reason taught	9.123	4	0.058
Q.10 Encouragement to teach more	1.674	4	0.795

a α = 0.05.

b Significant differences indicated with an asterisk (*).

**Table 7.** Differences among IARS areas.

**Question number**	**H**	***d_f_***	***p*^a^,^b^**
Q.3 Frequency of lessons	3.502	5	0.623
Q.4 Length of lessons	9.885	5	0.079
Q.5 Subject	2.524	5	0.773
Q.6 Teaching method	6.053	5	0.301
Q.7 Global/Local issues	6.645	5	0.248
Q.8 Where teacher obtained information	6.307	5	0.278
Q.9 Reason taught	3.619	5	0.605
Q.10 Encouragement to teach more	11.589	5	0.041*

a α = 0.05.

b Significant differences indicated with an asterisk (*).

**Table 8.** Subject most often used to teach about the environment.

**Subject**	**Grade**					**Area**						**Total**	**Total %**
	**1st**	**2nd**	**3rd**	**4th**	**5th**	**I**	**II**	**III**	**IV**	**V**	**VI**		
Science	17	17	27	29	26	74	9	5	14	12	2	116	57.7
Social sci./studies	9	8	6	11	3	26	5	1	2	2	1	37	18.4
Math	0	0	0	0	1	1	0	0	0	0	0	1	0.5
Reading	2	2	0	0	1	2	1	0	1	1	0	5	2.5
Writing	0	0	0	0	0	0	0	0	0	0	0	0	0.0
Separate topic	0	0	1	0	4	4	1	0	0	0	0	5	2.5
Interdisciplinary	11	10	5	7	4	25	1	2	7	1	1	37	18.4
N	39	37	39	47	39	132	17	8	24	16	4	201	100

**Table 9.** Method most often used to teach about the environment.

**Method**	**Grade**					**Area**						**Total**	**Total %**
	**1st**	**2nd**	**3rd**	**4th**	**5th**	**I**	**II**	**III**	**IV**	**V**	**VI**		
Discussion	36	35	41	42	34	129	15	5	20	16	3	188	88.3
Research	1	0	2	5	1	7	0	1	1	0	0	9	4.2
Going outside	2	1	1	0	2	2	2	0	2	0	0	6	2.8
Field trips	1	1	1	2	1	3	0	1	2	0	0	6	2.8
Exploring values	1	1	1	1	0	4	0	0	0	0	0	4	1.9
N	41	38	46	50	38	145	17	7	25	16	3	213	100

**Table 10.** Reasons why teachers chose to omit EE.

**Reasons**	**Grade**					**Area**						**Total**	**Total %**
	**1st**	**2nd**	**3rd**	**4th**	**5th**	**I**	**II**	**III**	**IV**	**V**	**VI**		
Not enough class time	0	2	2	4	1	7	2	0	0	0	0	9	47.4
Topics are unrelated	0	0	0	1	3	4	0	0	0	0	0	4	21.1
Do not know enough	0	0	0	1	0	1	0	0	0	0	0	1	5.3
District does not emphasize it	0	0	1	0	1	2	0	0	0	0	0	2	10.5
School does not emphasize it	0	0	0	0	0	0	0	0	0	0	0	0	0.0
Did not know a requirement	1	0	0	0	1	2	0	0	0	0	0	2	10.5
Other topics are more important	0	0	0	0	1	1	0	0	0	0	0	1	5.3
Not appropriate for grade level	0	0	0	0	0	0	0	0	0	0	0	0	0.0
Students not interested/concerned	0	0	0	0	0	0	0	0	0	0	0	0	0.0
N	1	2	3	6	7	17	2	0	0	0	0	19	100

**Table 11.** Reasons why teachers chose to include EE.

**Reasons**	**Grade**					**Area**						**Total**	**Total %**
	**1st**	**2nd**	**3rd**	**4th**	**5th**	**I**	**II**	**III**	**IV**	**V**	**VI**		
Req’d by IL law	0	0	1	2	0	1	0	0	1	1	0	3	1.5
Req’d by IL learning standards	5	8	8	10	3	20	5	3	3	3	0	34	17.3
Req’d by district curriculum	6	4	9	6	11	26	2	1	2	5	0	36	18.3
District emphasis	1	0	1	0	0	2	0	0	0	0	0	2	1.0
School emphasis	0	0	1	1	0	2	0	0	0	0	0	2	1.0
Personal interest	18	21	16	22	20	63	8	3	15	5	3	97	49.2
Students asked about environment	1	2	1	1	1	4	1	0	0	1	0	6	3.0
Think students are concerned	2	2	7	5	1	15	1	0	1	0	0	17	8.6
N	33	37	44	47	36	133	17	7	22	15	3	197	100

**Table 12.** Events that would encourage teachers to include more EE.

**Method**	**Grade**					**Area**						**Total**	**Total %**
	**1st**	**2nd**	**3rd**	**4th**	**5th**	**I**	**II**	**III**	**IV**	**V**	**VI**		
More emphasis from district	2	4	5	6	7	18	1	0	5	0	0	24	12.1
More emphasis from school	0	2	1	1	0	2	1	0	5	0	0	4	2.0
More in-service training	5	12	9	12	11	31	6	2	6	4	0	49	24.6
More planning time	9	6	9	10	8	26	6	2	4	2	2	42	21.1
More/easier access to resources	20	16	17	18	9	55	3	3	8	10	1	80	40.2
N	36	40	41	47	35	132	16	7	23	18	3	199	100

**Table 13.** Events that would encourage teachers to include EE.

**Method**	**Grade**					**Area**						**Total**	**Total %**
	**1st**	**2nd**	**3rd**	**4th**	**5th**	**I**	**II**	**III**	**IV**	**V**	**VI**		
More emphasis from district	1	1	1	1	4	8	0	0	0	0	0	8	42.1
More emphasis from school	0	0	0	0	2	1	1	0	0	0	0	2	10.5
More in-service training	0	0	0	1	1	2	0	0	0	0	0	2	10.5
More planning time	0	0	2	0	0	2	0	0	0	0	0	2	10.5
More/easier access to resources	0	1	0	3	1	4	1	0	0	0	0	5	26.3
N	1	2	3	5	8	17	2	0	0	0	0	19	100

## Footnotes

a IARSS divided the state into six Areas based on the Regional Offices of Education located throughout the state. Area I consists of Northeastern Illinois, Area II of Northwestern Illinois, Area III of West Central Illinois, Area IV of East Central Illinois, Area V of Southwestern Illinois, and Area VI of Southeastern and Southern Illinois.

b In lieu of a separate discussion section, the authors opted to combine the results and discussion sections to facilitate the understanding of the study’s implications.
